# Editorial: Intellectual Disability and Assistive Technology

**DOI:** 10.3389/fpubh.2019.00171

**Published:** 2019-07-02

**Authors:** Fleur Heleen Boot, Julia S. Louw, Hung Jen Kuo, Roy Chen

**Affiliations:** ^1^Department of Intellectual Disability Medicine, Maynooth University, Maynooth, Ireland; ^2^Department of Psychology, National University of Ireland Galway, Galway, Ireland; ^3^Department of Special Education and Counseling, California State University, Los Angeles, CA, United States; ^4^School of Rehabilitation Services and Counseling, University of Texas Rio Grande Valley Edinburg, Edinburg, TX, United States

**Keywords:** assistive technology, assistive products, intellectual disability, health inequity, cognitive functioning

People with intellectual disabilities (ID) need assistive technology (AT) to maintain and improve their levels of functioning and independence, which in turn promotes well-being. AT enhances the ability of people with cognitive limitations to participate in and integrate into an inclusive society. AT can also be used to better manage comorbidities, and people with ID have a higher prevalence of comorbidity compared to the general population. With the current growth of aging populations, the prevalence of older people with ID is likely to increase along with the demand for access to AT. Nevertheless, people with ID are disproportionately affected by disparities in healthcare services and in the acquisition of AT.

Although AT offers great opportunities for improving quality of life, it's people with ID who remain an underexplored focus for research and practice. This Research Topic compiles six articles that highlight the need, impact, and possibilities of AT for people with ID. Through the lens of multidisciplinary perspectives, the objective of the Research Topic is to facilitate a better understanding of the needs of people with ID. Specifically, it focuses on how to close the gap between services and outcomes and also to increase the inclusion of this population in society. The articles shed light on three broad themes related to AT. The first theme (O'Brolcháin; Boot et al.) offers perspectives on the importance of ethical considerations regarding ID and AT, and how the challenges that ID presents should be considered. The second theme (Lancioni et al.; Robb et al.; Lee et al.) reports on results and outcomes of three original empirical studies that include a smart-phone intervention, a computerized cognitive training method, and an assessment on cognitive functioning among children. The third theme (Ngomwa) illustrates the impact of policy on AT use in the context of Malawi, Africa.

The first article (O'Brolcháin) describes the right to autonomy and the associated risks of AT for people with ID. The article argues that AT can both promote and undermine autonomy, specifically in the areas of knowledge, authenticity, and liberty. Undermining the autonomy of people with ID is most pronounced in those with severe to profound ID, because it is difficult to determine their preferences and the degree of autonomy in these cases. Ethical oversight should be included when developing AT for people with ID, according to the author.

The second article (Boot et al.) provides insights into the value of including people with ID in global initiatives related to AT and improving access to AT. It focuses on the factors related to the need for and provisions of AT that are critical to serving people with ID and discusses ways to address these factors.

The third article, an empirical study (Lancioni et al.) demonstrates the use of smartphones to help people with ID and sensory impairments to perform daily living activities. In this study, each participant was given a smartphone, which was set up to deliver verbal or vibratory and visual reminders at the times when activities were planned and to present verbal or pictorial instructions for the steps of these activities. This intervention study is an example of how AT can be adapted and used for people with ID to enhance their independence.

The fourth article, an empirical study by Robb et al. examines the attitudes of parents to support the use of computerized cognitive training to improve executive functioning in children with neurodevelopmental disorders. Children often depend on parental support to use such computerized cognitive training. The findings show that parents see the potential of such training, especially to improve social skills, motor skills, cognitive skills, and quality of life.

In the fifth article, Lee et al. explain how interactive block games are used in relation to AT to assess children's cognitive functioning. Although many validated tests are available to assess cognitive functioning, these tests often have inherent problems such as high costs associated with testing, limited availability of appropriately trained clinicians, and susceptibility to human error. This study compares the use of the interactive block games method with the traditional intellectual assessments.

In the sixth article, Ngomwa provides mini reviews on ID and improved access to AT in Malawi. The article presents the barriers faced by Malawian people with ID and examines the policies and legislation that can positively influence access to AT for this group. The article also discusses and recommends ways to remove barriers and enhance access to AT.

The functional limitations of ID vary greatly from one individual to another. The uncertainty of functional limitations is further complicated by the high comorbidity rate. This makes managing the activities of daily living more challenging, and also increases the cost for the professional health care. With the increased prevalence rate of the ID population and the need for long-term care, it is anticipated that the challenges will only become more serious. AT has provided some promising results. It is our hope that this Research Topic will shed some lights on this important but often overlooked topic, and thus spark more discussion and support for the ID population and AT solutions.

## Author Contributions

JL, RC, HK, and FB: substantial contributions to the conception and design of the work, drafting the work, revising the work critically for important intellectual content, final approval of the version to be published, and agreement to be accountable for all aspects of the work in ensuring questions related to the accuracy or integrity of any part of the work are appropriately investigated and resolved. The authors alone are responsible for the views expressed in this article and they do not necessarily represent the views, decisions or policies of the institutions with which they are affiliated.

### Conflict of Interest Statement

The authors declare that the research was conducted in the absence of any commercial or financial relationships that could be construed as a potential conflict of interest.

